# Editorial: Diverse functions of drug transporters

**DOI:** 10.3389/fphar.2025.1697775

**Published:** 2025-10-06

**Authors:** Jeffry C. Granados, Kit Wun Kathy Cheung, Bhagwat Prasad

**Affiliations:** ^1^ Translational Pharmacokinetics and Pharmacodynamics, Genentech, Inc., South SanFrancisco, CA, United States; ^2^ Clinical Pharmacology, Genentech, Inc., South SanFrancisco, CA, United States; ^3^ Translational and Clinical Pharmacology, Cincinnati Children’s Hospital Medical Center, Cincinnati, OH, United States

**Keywords:** drug transporter, membrane protein, substrate, inhibitor, competitive interaction

Drug transporters are a subset of around 10–20 membrane transporters that are mainly known for their role in the absorption, distribution, metabolism, and excretion (ADME) of small molecule drugs. While these transporters exhibit diverse mechanisms of action and tissue expression patterns, their shared feature share is multi-specificity - the ability to recognize and transport dozens of structurally unique compounds, with each transporter having its own set of substrates/inhibitors. Driven by regulatory requirements, research exploring the multi-specificity of these proteins has predominantly focused on pharmaceutically related molecules. Consequently, while informative, this emphasis has reinforced the potentially narrow perception of these proteins as exclusively “drug” transporters.

Indeed, several studies have demonstrated that the specificity of these drug transporters is not limited to drugs. Endogenous metabolites, natural products, and food products have been shown to interact with these transporters, which begs the question: What else are drug transporters capable of (Nigam, 2015)? Better understanding the full profile of substrates/inhibitors of drug transporters can help de-risk potential competitive interactions occurring between multiple different small molecules. This also enables us to understand the physiological roles of transporters and their links to genetic disorders as well as identify biomarkers for specific transporters. In particular, biomarkers can be leveraged for drug-drug interaction (DDI) risk prediction during Phase I studies and for understanding inter-individual variability. This special edition aims to further probe the multi-specificity of drug transporters, with a particular focus on non-pharmaceutical molecules. In addition to identifying new substrates/inhibitors, we are also interested in better understanding the physicochemical interaction between small molecules and their associated transporters.

The distinction between drugs and natural products is unclear, as many “pharmaceutical” products are derived from or inspired by natural products. For example, mycophenolate, an immunosuppressant, is derived from fungi. In a manuscript by Mao et al., the authors demonstrate that a derivative of mycophenolate mofetil, mycophenolic acid glucuronide (MPAG), interacted with the drug transporters OATP1B1 and OATP1B3 via competitive interaction with cyclosporine A, a fungal derived product, *in vitro*. They also showed that MPAG was transported by OAT3. The authors also investigated the natural product salvia miltiorrhiza, which has been used to treat cardiovascular disease, and found inhibition of OATP and OAT3 activity that impacted MPAG uptake. This work is a clear example of drug-natural product interactions as a result of competition at multi-specific drug transporters.

In Liu et al., the authors explore the natural products betulinic acid and the related molecule, 23-hydroxybetulinic acid (23-HBA). These natural products have demonstrated potential for therapeutic benefit, mainly as cytotoxic agents against tumor cells, but little is known about their interactions with drug transporters. The authors first show that 23-HBA increased intracellular levels of classic P-gp substrates, suggesting that the compound is an inhibitor of P-gp. In addition to *in vitro* assays to establish the interaction between P-gp and 23-HBA, the authors also leveraged the protein structure of P-gp to better understand the differences in binding between the structurally similar betulinic acid and 23-hydroxybetulinic acid. Docking simulations revealed that minor differences in structure can lead to different interactions within the P-gp binding pocket, which helps partly explain, from a structural perspective, a potential mechanism for the multi-specificity of P-gp.

Beyond natural products, drug transporters also have roles in transporting endogenous compounds. Wen et al. investigated the function of OATP1A2 in astrocytes and found that, *in vitro*, OATP1A2 appears to be an uptake transporter for amyloid beta peptides, as classic OATP inhibitors decreased the intracellular levels of amyloid beta in the cells. Amyloid beta is neither a drug nor natural product, which highlights that drug transporters may have the capacity to handle some peptides. Indeed, multiple manuscripts have demonstrated that P-gp plays a meaningful role in the handling of amyloid beta (Wang et al., 2016; Chai et al., 2020), suggesting that some peptides may have distinct uptake and efflux transporters.

Finally, the manuscript by AbdulHameed et al., leverages the existing substrate/inhibitor data on 12 well-studied drug transporters, including OAT1 and P-gp. By applying machine learning techniques based on the chemical structures of the known interacting molecules, the authors were able to generate a fast, efficient tool to predict potential substrates and inhibitors for each transporter. Interestingly, while much of the data comes from drugs, the model itself is agnostic to the types of compounds and can be applied to predict whether natural products or endogenous metabolites interact with specific transporters. Furthermore, the authors generated an accessible web-based application that allows users from different fields to explore the chemical space associated with individual transporters (https://monstrous.bhsai.org/). This tool allows for easy virtual screening and can help generate hypotheses for whether non-pharmaceutical molecules are substrates or inhibitors of drug transporters.

In summary, the combined articles demonstrate that drug transporters play diverse and essential roles in pharmacokinetics, pharmacodynamics, and drug safety by regulating the absorption, distribution, and elimination of drugs, natural products, and endogenous compounds. Beyond their canonical function in drug disposition, emerging research highlights their roles in drug–natural product interactions, tissue-specific drug targeting, and the modulation of cellular signaling pathways. For example, transporters like OATP1B1 and P-gp influence hepatic and brain drug distribution, respectively, while SLC transporters impact nutrient and metabolite fluxes relevant to drug action. The publications included in this issue exemplify the multifaceted roles of transporters ranging from small molecule natural products to amyloid beta peptides, as well as novel techniques leveraging molecular and protein structures, offering fresh insights into their expanding relevance in drug development and personalized medicine.
